# Augmentation of cognitive-behavioural therapy for obsessive-compulsive and anxiety disorders: a protocol for a systematic review and meta-analysis

**DOI:** 10.1136/bmjopen-2024-090431

**Published:** 2024-11-01

**Authors:** Jonathan Torbecke, Till Langhammer, Lisa Mewes, Ulrike Lueken, Johannes Caspar Fendel

**Affiliations:** 1 Department of Psychology, Humboldt University of Berlin, Berlin, Germany; 2 Partner Site Berlin/Potsdam, German Center for Mental Health (DZPG), Potsdam, Germany; 3 Department of Psychosomatic Medicine and Psychotherapy, Medical Faculty, Medical Center, University of Freiburg, Freiburg im Breisgau, Germany

**Keywords:** Anxiety disorders, Systematic Review, Meta-Analysis

## Abstract

**Abstract:**

**Introduction:**

While cognitive-behavioural therapy (CBT) for obsessive-compulsive disorder (OCD) and anxiety disorders (ADs) has been proven to be effective and is commonly recommended, a considerable proportion of patients remain symptomatic, do not respond to treatment or discontinue it. Thus, augmentation strategies aimed at enhancing CBT outcomes are essential to reduce the burden of OCD and ADs on patients and society. Various augmentation strategies for CBT in OCD and ADs have been investigated, yet it remains unclear if they show robust beneficial effects beyond first-line CBT. With this systematic review and meta-analysis, we will provide an overview and critically assess the efficacy of non-pharmacological augmentation strategies in addition to first-line CBT treatment for symptom reduction, response rates and dropout rates in individuals with OCD or ADs.

**Methods and analysis:**

We will screen PubMed, Embase, PsycArticles, PsycInfo, CINAHL, PSYNDEX and Cochrane Register of Controlled Trials without restrictions on publication dates or languages. Additionally, forward, and backward searches of included studies and systematic reviews will be conducted. Two reviewers will independently screen the studies, extract data and assess the methodological quality of the studies. We will exclusively include randomised controlled trials. The primary outcomes will be symptom severity and response rates. Dropout rates will serve as a secondary outcome. Moreover, we will provide a narrative review of the results. We will use subgroup and meta-regression analyses to identify potential moderators and sources of between-study heterogeneity. We will use the Grading of Recommendations Assessment, Development and Evaluation system to assess the overall quality of evidence.

**Ethics and dissemination:**

Ethical approval is not required. Results will be published in a peer-reviewed journal.

**PROSPERO registration number:**

CRD42024561027.

**STRENGTHS AND LIMITATIONS OF THIS STUDY**

- Including only randomised controlled trials (RCTs) reduces bias and enhances the reliability of our findings.
- We will use subgroup and meta-regression analyses to explore potential moderators and heterogeneity, strengthening the robustness of our results.
- We evaluate multiple key outcomes—symptom reduction, response rates and dropout rates—to broaden the scope of our analysis.
- Adherence to Preferred Reporting Items for Systematic Review and Meta-Analysis Protocols guidelines ensures transparency and methodological rigour.
- Limiting the review to RCTs may reduce generalisability by excluding insights from other study designs.

## Introduction

### Rationale

 Obsessive-compulsive disorder (OCD) and anxiety disorders (ADs) are widespread, often persistent and functionally impairing mental disorders with severe implications for patients’ quality of life and the healthcare system in general.^1^,^6^ OCD affects 0.7%–1.2% of the adult population considering the 12-month prevalence, and between 2.3% and 3.5% taking into account the lifetime prevalence.^6^,^8^ ADs represent the most pervasive group of mental disorders, with a 12-month prevalence ranging between 8.4% and 21.3% and a lifetime prevalence of up to 33.7%.^6 9^ Individuals with OCD or ADs share similar symptoms; both often experience recurring fearful thoughts, and exhibit avoidant and reassuring behaviours.^10^ These symptomatic similarities lead to similar first-line treatments. Considering these similarities, focusing on OCD and ADs together might provide valuable insights when studying and refining treatments.

Besides serotonin reuptake inhibitors (SSRIs), cognitive-behavioural therapy (CBT) is often referred to as the treatment of choice for OCD and ADs, with exposure methods and cognitive restructuring methods being applicable and commonly used to treat both disorders.^11^ In meta-analyses, CBT revealed large effects for OCD compared with waitlist control and placebo conditions,^12 13^ as well as large effects for ADs compared with waitlist and small to moderate effects compared with care-as-usual or pill placebo conditions.^14^ Moreover, CBT shows stable moderate effects on anxiety symptoms within 12 months after treatment completion.^15^ In line with these findings, national healthcare guidelines for the treatment of OCD and ADs recommend CBT as a first-line treatment besides SSRIs.^16^,^18^

However, not all patients benefit from CBT and many patients with OCD or ADs continue to experience considerable symptoms even after treatment. This is problematic since greater symptom severity is associated with reduced quality of life and increased functional impairment in OCD-patients^19^ as well as with reduced quality of life, higher comorbidity rates and greater clinical burden in patients with ADs.^20 21^ A substantial proportion of patients do not show a clinically meaningful response to CBT, with a recent meta-analysis reporting response rates around 38%–43% for OCD and 28%–41% for ADs, varying by the CBT-method used.^22^ While methodological differences (eg, regarding the definition of response) across studies contribute to varying rates, the issue of non-response appears more substantial than commonly recognised.^23^ Similarly, estimates of patients discontinuing CBT range from around 11%–19% for OCD^13 24 25^ and 15%–20% across ADs.^25 26^ This poses a major problem, as the majority of OCD patients discontinuing CBT is unlikely to experience clinically significant benefits.^24^ Furthermore, attending more CBT sessions has been shown to reduce both symptoms and functional disability among AD patients.^27^ Thus, it is crucial to investigate why symptoms persist and why some patients do not respond or discontinue treatment. Based on these findings, treatment approaches need to be re-evaluated and improved accordingly. While machine learning approaches are promising in predicting treatment outcomes of psychotherapy,^28^,^30^ recent research has also focused on the enhancement of first-line treatment efficacy through separate additional treatment components, known as augmentation strategies.

A variety of augmentation strategies for CBT targeting OCD and ADs have been explored. Besides pharmacological strategies (like D-cycloserine or SSRIs), studies focused on non-pharmacological augmentation such as attention bias modification (ABM), transcranial direct current stimulation, aerobic exercise, interpretation training, family involvement or motivational interviewing. First meta-analytical findings suggest that integrating CBT with psychosocial augmentation strategies, such as involving family members or implementing motivational interviewing, is beneficial to further reduce symptoms of OCD.^31^ The improved efficacy through these augmentation strategies beyond CBT was most pronounced in trials involving individuals with heightened severity of OCD symptoms at baseline and when administered separately from the CBT sessions.^31^ Involving relatives of OCD patients in family therapy or groups likely improves family functioning (eg, by reducing accommodation behaviours) enhancing the efficacy of CBT.^32 33^ Motivational interviewing might enhance CBT outcomes by addressing patients’ ambivalence about treatment, boosting their self-efficacy, helping them confront anxiety during exposure and response prevention exercises, clarifying the long-term relief from avoiding rituals and supporting consistent completion of homework assignments.^34 35^ While suggestions exist, the precise ways these strategies augment CBT are not yet fully understood. Considering that the average effect of CBT is already moderate to high, despite a rather limited response rate, it is particularly important to test for improvements in response rate in addition to effect sizes. Furthermore, recent research^36 37^ and advances in the field have refined the conceptualisation of CBT augmentation for OCD patients. Regarding ADs, a meta-analysis focusing on ABM in addition to CBT showed small effects on clinician-rated symptoms, while effects on self-rated and parental-rated symptoms remained non-significant.^38^ Hang *et al*^38^ highlight that models by Beck and Clark^39^ or Mogg and Bradley^40^ both assume bottom-up (stimulus-driven) and top-down (cognitively driven) processes to contribute to anxiety. Therefore, combining CBT and ABM is promising because it involves both cognitive processes. Interestingly, this meta-analysis suggests that using ABM with CBT in the same session is more effective than applying them separately.^38^ To the best of our knowledge systematic reviews on different non-pharmacological augmentation in addition to CBT for ADs do not exist. As outlined, early meta-analytical results focused on symptom reduction, leaving response and dropout rates unexamined.^31 38^ Considering them might clarify who benefits from augmentation strategies: are augmentation strategies only effective for patients who would already complete the first-line treatment and show a clinically meaningful response, or can they help those who would otherwise discontinue or not respond? Although some studies report promising results for OCD and ADs,^3235^,^37 41 42^ the efficacy of non-pharmacological strategies augmenting CBT for these patients with similar symptoms (eg, avoidant and reassuring behaviours) and first-line treatment (eg, exposure methods) has not yet been jointly investigated. Given that similar psychotherapeutic methods aid both OCD and AD patients, it appears reasonable that augmentation strategies with similar characteristics might be beneficial to both. In summary, we plan to update and extend existing systematic reviews on augmentation strategies in addition to CBT, focusing on non-pharmacological strategies for OCD and ADs.

### Objectives

We will provide healthcare policymakers, practitioners and researchers with a thorough overview of the existing state of knowledge within the expanding domain of non-pharmacological augmentation in the first-line treatment (CBT) of OCD and ADs. We aim to systematically review and meta-analyse the efficacy of non-pharmacological augmentation strategies administered in addition to CBT for symptom reduction, improvement of response rates (primary outcomes) and lower dropout rates (secondary outcome) in individuals diagnosed with OCD and ADs. Hence, our research aims to answer the following questions: (1) what characterises existing non-pharmacological augmentation strategies in terms of modality, content, mechanisms addressed, application methods and intended health implications?, (2) do non-pharmacological augmentation strategies improve symptom severity, response rates and dropout rates? and (3) which factors moderate the effects and explain sources of between-study heterogeneity (eg, patients’ diagnoses, treatment resistance at baseline, CBT method used, age, proportion of female participants, proportion of medicated participants, dosage of both treatment components (CBT and augmentation), different delivery formats and characteristics of the specific augmentation strategy)?

## Methods

We prepared this protocol in adherence to the Preferred Reporting Items for Systematic Review and Meta-Analysis Protocols (PRISMA-P).^43^ A completed PRISMA-P checklist including recommended items for a systematic review protocol is available in online supplemental material. The subsequent systematic review and meta-analysis report will conform to the guidelines outlined in the Preferred Reporting Items for Systematic Reviews and Meta-Analyses (PRISMA).^44^ This study protocol has been registered on the International Prospective Register of Systematic Reviews platform. The study is planned to commence in September 2024 and conclude in January 2025.

### Eligibility criteria

#### Population

Eligible study samples will include individuals who meet the diagnostic criteria of the Diagnostic and Statistical Manual of Mental Disorders or the International Classification of Diseases for OCD, panic disorder, agoraphobia, social anxiety disorder, generalised anxiety disorder or specific phobia. We will include samples receiving no or consistent psychotropic medication. Therefore, samples with changes in their psychotropic medication during the trial will be excluded. We will also exclude trials involving non-clinical samples or undiagnosed patients. The inclusion and exclusion criteria are listed in table 1.

**Table 1:** Inclusion and exclusion criteria

Criterion	Inclusion criteria	Exclusion criteria
Population	Patients diagnosed with OCD or AD receiving CBT and no or consistent psychotropic medication	Non-clinical samples, undiagnosed patients, patients with changes in their psychotropic medication during the study
Intervention	Interventions augmenting CBT with non-pharmacological treatments	Interventions augmenting CBT with pharmacological treatments
Comparator	CBT only or CBT with active control condition (augmentation placebo)	Augmentation strategy only
Outcomes	OCD or AD symptoms measured with validated self-reports and/or clinician rated quantitative measures at postintervention and response rates (primary outcomes), dropout rates (secondary outcome)	OCD or AD symptoms measured with self-reports without validation
Study	RCTs (with and without active control condition in addition to CBT)	Non-randomised trials, including non-controlled before–after studies, case–control studies, single case studies, clinical case studies, qualitative studies
Language	All languages	None
Publication date	All dates	None

ADanxiety disorderCBTcognitive-behavioural therapyOCDobsessive-compulsive disorderRCTrandomised clinical trial

#### Interventions

We will include all non-pharmacological augmentation strategies that are administered in addition to a first-line CBT. Pharmacological augmentation strategies will be excluded. We define augmentation strategy as any non-pharmacological intervention ‘delivered prior to, concurrently, or after a first line […] treatment, where the focus of the augmentation was to improve […] symptoms and/or improve readiness for treatment, engagement, or retention in the first-line treatment’.^45^

#### Study designs and comparators

We will include randomised controlled trials (RCTs) comparing CBT with CBT plus an augmentation strategy. Comparison conditions may encompass an active control component in addition to CBT or not.

#### Outcome measures

The first primary outcome will be symptom severity of OCD or AD at postintervention, assessed using validated measures. If more than one measure of symptoms is reported, we will prioritise the outcome defined as primary outcome. If unclear, we will prioritise clinician-rated (eg, Hamilton Anxiety Rating Scale^46^ or Yale-Brown Obsessive Compulsive Scale^47^) over self-reported measures. Furthermore, as a second primary outcome, we will assess differences in response rates using symptom reduction in OCD and AD. We will define patients as responders if they achieve at least a 50% reduction^48 49^ in AD symptoms or a 35% reduction in OCD symptoms^50^ from baseline to postmeasurement. Following Cuijpers *et al*,^22^ we will estimate response rates using the validated method by Furukawa *et al*.^51^ This method addresses the high heterogeneity in response definitions across studies,^23^ facilitating a more standardised comparison. To verify the robustness of our results, we will conduct a sensitivity analysis using the study authors’ definitions of response. The secondary outcome will be dropout rates, specified as reported by the authors. If it is unspecified, we will contact the authors and calculate it based on the most used definition of the other studies included in this review.

### Search strategy

We will systematically search PubMed, Embase via OVID, EBSCOhost (including PsycArticles, PsycInfo, CINAHL and PSYNDEX) and Cochrane Register of Controlled Trials. We will apply a sensitivity-optimised search strategy without restrictions regarding publication languages or dates. The search terms will be related to (1) obsessive-compulsive or ADs, (2) CBT, (3) augmentation and (4) RCT, using text words and controlled vocabulary (including MeSH terms). All search strategies are provided in online supplemental material. In addition, we will conduct backward citation searches for all included studies and relevant reviews and meta-analyses, alongside forward citation searches. If access to the full text of references is unavailable, we will contact the authors of these studies and await their response for 2 months. To include potential unpublished data, we will contact the corresponding authors of all included studies to inquire whether they have or know of any additional unpublished data. Furthermore, we will reach out to authors of pre-registrations (eg, registered on clinicaltrials.gov) and study protocols identified during the search.

### Study selection

Two reviewers (JT and TL) will independently conduct a software-based screening of titles and abstracts. Full texts will be obtained if at least one reviewer considers that an article meets the inclusion criteria. Subsequently, both reviewers will independently assess the eligibility of individual references through comprehensive full-text screening. All reasons for exclusions will be documented. In adherence to the PRISMA guidelines,^44^ we will outline the literature search and study selection process in a flowchart.

### Data extraction

Two reviewers (JT and LM) will autonomously extract information from eligible studies using a standardised template, piloting and modifying it as needed. We will resolve disagreements through discussion and consensus. We will extract information on (1) the study: authors, publication year, country, type of control, CBT methods used, inclusion and exclusion criteria, (2) the sample: sample size at pre-, post- and follow-up measurement, age, sex, ethnicity, diagnosis, treatment resistance at baseline, comorbidity, medication, (3) the intervention and comparator: type of implemented augmentation strategy, delivery format, duration (timespan), duration of an average session, number of sessions, frequency of sessions, schedule (prior to, concurrently or after CBT), use of measurement tools for intervention integrity including adherence to the protocol and to assess the clinical and programme experience of the facilitating therapist, adverse events and (4) the outcomes: measure of symptom severity, means and SDs of symptom severity of OCD and ADs at pre-, post- and follow-up measurement, response rates (including the definition used in the respective study and absolute numbers of response/non-response) and dropout rates (including the definition used in the respective study and absolute numbers of completing/discontinuing patients). The extracted data will be entered into the statistical software RStudio.^52^ In instances of incomplete data, we will reach out to the respective study authors and await their response for a duration of 2 months.

### Risk of bias and quality assessment in individual studies

Two reviewers (JT and TL) will independently assess the studies for their risk of bias. Cases of discrepancy will be resolved through discussion and consensus. Any remaining discrepancies will be addressed and resolved through discussion with a third reviewer (JCF). Bias of included RCTs will be evaluated using the second version of the Cochrane risk-of-bias assessment tool for randomised trials (RoB2).^53^ RoB2 assesses bias in five domains: (1) bias arising from the randomisation process, (2) deviations from intended interventions, (3) missing outcome data, (4) measurement of the outcome and (5) selection of the reported result. Within each domain, the risk of bias is rated as ‘low risk of bias’, ‘some concerns’ or ‘high risk of bias’. In addition to the domain-specific judgements, a corresponding overall risk-of-bias for each outcome will be derived.

### Risk of bias across studies

To evaluate a potential publication bias we will visually scrutinise the funnel plots for asymmetry,^54 55^ compute Rosenthal’s fail-safe N,^56^ conduct Egger’s tests^57^ and conduct the ‘trim and fill procedure’.^58^ We will use the Grading of Recommendations Assessment, Development and Evaluation (GRADE) approach^59^ to assess the overall quality of evidence. With GRADE the overall quality of evidence is rated as ‘high’, ‘moderate’, ‘low’ or ‘very low’ for each outcome.

### Data synthesis

#### Quantitative and narrative synthesis approach

Anticipating diverse characteristics in augmentation strategies, which might result in noteworthy heterogeneity, we will conduct random-effects meta-analyses. For the primary outcomes, we will calculate pooled Hedges’ g^60^ as a measure of the standardised mean difference in OCD/AD symptom severity at postintervention as well as the pooled ORs for differences in response rates. For the secondary outcome, we will calculate pooled ORs for differences in dropout rates. We will calculate all effect sizes along with their 95% CIs and corresponding p values. Sensitivity analyses will be performed regarding outliers, follow-up, study sample (intention-to-treat/completing participants) and risk of bias. We will identify outliers using the ‘non-overlapping CI approach’.^61^ We will assess between-study heterogeneity among the included studies using the Q-test and I² statistics.^62^ According to the Cochrane Handbook, I² values will be interpreted as unimportant (I²<40%), moderate (30%–60%), substantial (50%–90%) or considerable heterogeneity (75%–100%).^63^ Moreover, we will calculate the prediction interval, as a range into which the true effect size of 95% of all populations will fall.^64^ Sources of between-study heterogeneity will be explored by subgroup and meta-regression analyses. Subgroup analyses will be conducted if a total of ten studies are available, with each subgroup requiring a minimum of three studies.^65^ We prespecify subgroup analyses on patients’ diagnoses, treatment resistance at baseline, CBT method used, age, different delivery formats and characteristics of augmentation approaches. We will refine subgroup criteria and might add post-hoc analyses as data on augmentation strategy characteristics or delivery formats become available throughout the process. Meta-regression analyses will be performed if at least 10 studies are available.^63^ We prespecify meta-regression analyses to evaluate how the proportion of female participants, proportion of medicated participants and dosage of both treatment components (CBT and augmentation) may moderate the effect. If the number of studies available for the subgroup and meta-regression analyses is insufficient (ie, fewer than 10 times the number of subgroup and meta-regression analyses), they will be conducted as exploratory and hypothesis-generating, rather than conclusive. This limitation will be carefully considered when interpreting the results, and the findings will be framed as preliminary insights to guide future research. Finally, we will conduct a narrative synthesis of the study characteristics. We will provide a comprehensive presentation of the results in all relevant areas using a ‘Summary of findings’ table. All analyses will be conducted in RStudio.^52^

#### A priori power calculation

We conducted an a priori power analysis to evaluate the feasibility of detecting clinically meaningful effects within the expected constraints of our meta-analysis, including the small number of studies, limited sample sizes and high heterogeneity. This allows us to better understand the potential limitations of our findings and ensures that our interpretations remain appropriately cautious. To address the difficulties of estimating precise values in this emerging field we decided to use conservative parameters. We aimed to detect a clinically relevant effect size of 0.2, setting the significance level at 0.05 for a one-sided test. Anticipating large heterogeneity across studies, we used a random-effects model and estimated the between-study variance following the guidelines by Hedges and Pigott^66^ as suggested by Harrer *et al*.^61^ We expected to include at least 10 studies, each with at least 20 participants per group. The power analysis, conducted using the ‘power.analysis’ function from the dmetar package^67^ in RStudio,^52^ yielded an estimated power of 0.29. This suggests that, given these conservative parameters, the meta-analysis may have limited ability to detect small effects, reinforcing the need to interpret the findings cautiously.

### Patient and public involvement

The study design as well as conduct, reporting or dissemination plans will not incorporate input or involvement from patients or the public.

## Ethics and dissemination

Ethical clearance is deemed unnecessary for this study as it involves the analysis of published studies and does not include the collection of primary data or direct involvement of human participants. The results will undergo dissemination through a peer-reviewed scientific journal.

## supplementary material

10.1136/bmjopen-2024-090431 online supplemental file 1

10.1136/bmjopen-2024-090431 online supplemental file 2

## References

[1] Baxter AJ, Vos T, Scott KM (2014). The global burden of anxiety disorders in 2010. Psychol Med.

[2] Markarian Y, Larson MJ, Aldea MA (2010). Multiple pathways to functional impairment in obsessive-compulsive disorder. Clin Psychol Rev.

[3] Mendlowicz MV, Stein MB (2000). Quality of life in individuals with anxiety disorders. Am J Psychiatry.

[4] Olatunji BO, Cisler JM, Tolin DF (2007). Quality of life in the anxiety disorders: A meta-analytic review. Clin Psychol Rev.

[5] Tolin DF, Gilliam CM, Dufresne D, Stein DJ, Hollander E, Rothbaum BO (2010). Textbook of anxiety disorders.

[6] Wittchen HU, Jacobi F, Rehm J (2011). The size and burden of mental disorders and other disorders of the brain in Europe 2010. Eur Neuropsychopharmacol.

[7] Angst J, Gamma A, Endrass J (2004). Obsessive-compulsive severity spectrum in the community: prevalence, comorbidity, and course. Eur Arch Psychiatry Clin Neurosci.

[8] Ruscio AM, Stein DJ, Chiu WT (2010). The Epidemiology of Obsessive-Compulsive Disorder in the National Comorbidity Survey Replication. Mol Psychiatry.

[9] Bandelow B, Michaelis S (2015). Epidemiology of anxiety disorders in the 21st century. Dialogues Clin Neurosci.

[10] American Psychiatric Association (2013). Diagnostic and statistical manual of mental disorders: DSM-5.

[11] McKay D, Norcross JC, VandenBos GR, Freedheim DK APA handbook of clinical psychology: Psychopathology and health.

[12] Olatunji BO, Davis ML, Powers MB (2013). Cognitive-behavioral therapy for obsessive-compulsive disorder: a meta-analysis of treatment outcome and moderators. J Psychiatr Res.

[13] Öst L-G, Havnen A, Hansen B (2015). Cognitive behavioral treatments of obsessive-compulsive disorder. A systematic review and meta-analysis of studies published 1993-2014. Clin Psychol Rev.

[14] Cuijpers P, Cristea IA, Karyotaki E (2016). How effective are cognitive behavior therapies for major depression and anxiety disorders? A meta-analytic update of the evidence. World Psychiatry.

[15] van Dis EAM, van Veen SC, Hagenaars MA (2020). Long-term Outcomes of Cognitive Behavioral Therapy for Anxiety-Related Disorders: A Systematic Review and Meta-analysis. JAMA Psychiatry.

[16] (2022). Deutsche Gesellschaft für Psychiatrie und Psychotherapie, Psychosomatik und Nervenheilkunde e.V. (DGPPN). S3-Leitlinie Zwangsstörungen – Langversion. https://register.awmf.org/de/leitlinien/detail/038-017.

[17] National Collaborating Centre for Mental Health (UK) (2006). Obsessive-Compulsive Disorder: Core Interventions in the Treatment of Obsessive-Compulsive Disorder and Body Dysmorphic Disorder.

[18] (2021). Deutsche Gesellschaft für Psychosomatische Medizin und Ärztliche Psychotherapie e.V. (DGPM). S3 - Leitlinie Behandlung von Angststörungen, Version 2. https://register.awmf.org/de/leitlinien/detail/051-028.

[19] Huppert JD, Blair Simpson H, Nissenson KJ (2009). Quality of Life and Functional Impairment in Obsessive-Compulsive Disorder: A comparison of patients with and without comorbidity, patients in remission, and healthy controls. Depress Anx.

[20] Wilmer MT, Anderson K, Reynolds M (2021). Correlates of Quality of Life in Anxiety Disorders. Rev of Rec Res Curr Psychiatry Rep.

[21] Penninx BW, Pine DS, Holmes EA (2021). Anxiety disorders. The Lancet.

[22] Cuijpers P, Miguel C, Ciharova M (2024). Absolute and relative outcomes of psychotherapies for eight mental disorders: a systematic review and meta-analysis. World Psychiatry.

[23] Gloster AT, Rinner MTB, Ioannou M (2020). Treating treatment non-responders: A meta-analysis of randomized controlled psychotherapy trials. Clin Psychol Rev.

[24] Leeuwerik T, Cavanagh K, Strauss C (2019). Patient adherence to cognitive behavioural therapy for obsessive-compulsive disorder: A systematic review and meta-analysis. J Anxiety Disord.

[25] Hans E, Hiller W (2013). A meta-analysis of nonrandomized effectiveness studies on outpatient cognitive behavioral therapy for adult anxiety disorders. Clin Psychol Rev.

[26] Fernandez E, Salem D, Swift JK (2015). Meta-analysis of dropout from cognitive behavioral therapy: Magnitude, timing, and moderators. J Consult Clin Psychol.

[27] Glenn D, Golinelli D, Rose RD (2013). Who Gets the Most Out of Cognitive-Behavioral Therapy for Anxiety Disorders. J Consult Clin Psychol.

[28] Chavanne A, Meinke C, Langhammer T (2023). Individual-Level Prediction of Exposure Therapy Outcome Using Structural and Functional MRI Data in Spider Phobia: A Machine-Learning Study. Depress Anxiety.

[29] Hilbert K, Kunas SL, Lueken U (2020). Predicting cognitive behavioral therapy outcome in the outpatient sector based on clinical routine data: A machine learning approach. Behav Res Ther.

[30] Hilbert K, Jacobi T, Kunas SL (2021). Identifying CBT non-response among OCD outpatients: A machine-learning approach. Psychother Res.

[31] Guzick AG, Cooke DL, Gage N (2018). CBT-Plus: A meta-analysis of cognitive behavioral therapy augmentation strategies for obsessive-compulsive disorder. J Obsessive Compuls Relat Disord.

[32] Peris TS, Rozenman MS, Sugar CA (2017). Targeted Family Intervention for Complex Cases of Pediatric Obsessive-Compulsive Disorder: A Randomized Controlled Trial. J Am Acad Child Adolesc Psychiatry.

[33] Thompson-Hollands J, Abramovitch A, Tompson MC (2015). A randomized clinical trial of a brief family intervention to reduce accommodation in obsessive-compulsive disorder: a preliminary study. Behav Ther.

[34] Merlo LJ, Storch EA, Lehmkuhl HD (2010). Cognitive behavioral therapy plus motivational interviewing improves outcome for pediatric obsessive-compulsive disorder: a preliminary study. Cogn Behav Ther.

[35] Meyer E, Souza F, Heldt E (2010). A randomized clinical trial to examine enhancing cognitive-behavioral group therapy for obsessive-compulsive disorder with motivational interviewing and thought mapping. Behav Cogn Psychother.

[36] Wolters LH, Hagen A, Beek V op de (2021). Effectiveness of an online interpretation training as a pre-treatment for cognitive behavioral therapy for obsessive-compulsive disorder in youth: A randomized controlled trial. J Obsessive Compuls Relat Disord.

[37] Katz DE, Rector NA, McCabe RE (2023). The effect of aerobic exercise alone and in combination with cognitive behavioural therapy on obsessive compulsive disorder: A randomized control study. J Anxiety Disord.

[38] Hang Y, Xu L, Wang C (2021). Can attention bias modification augment the effect of CBT for anxiety disorders? A systematic review and meta-analysis. Psychiatry Res.

[39] Beck AT, Clark DA (1997). An information processing model of anxiety: Automatic and strategic processes. Behav Res Ther.

[40] Mogg K, Bradley BP (2016). Anxiety and attention to threat: Cognitive mechanisms and treatment with attention bias modification. Behav Res Ther.

[41] Cobb AR, O’Connor P, Zaizar E (2021). tDCS-Augmented in vivo exposure therapy for specific fears: A randomized clinical trial. J Anxiety Disord.

[42] Zainal NH, Hellberg SN, Kabel KE (2023). Cognitive behavioral therapy (CBT) plus attention bias modification (ABM) reduces anxiety sensitivity and depressive symptoms in panic disorder: A pilot randomized trial. Scand J Psychol.

[43] Shamseer L, Moher D, Clarke M (2015). Preferred reporting items for systematic review and meta-analysis protocols (PRISMA-P) 2015: elaboration and explanation. BMJ.

[44] Page MJ, McKenzie JE, Bossuyt PM (2021). The PRISMA 2020 statement: an updated guideline for reporting systematic reviews. Syst Rev.

[45] Metcalf O, Stone C, Hinton M (2020). Treatment augmentation for posttraumatic stress disorder: A systematic review. Clin Psychol Sci Pract.

[46] HAMILTON M (1959). The assessment of anxiety states by rating. Br J Med Psychol.

[47] Goodman WK, Price LH, Rasmussen SA (1989). The Yale-Brown Obsessive Compulsive Scale. I. Development, use, and reliability. Arch Gen Psychiatry.

[48] Pittig A, Heinig I, Goerigk S (2021). Efficacy of temporally intensified exposure for anxiety disorders: A multicenter randomized clinical trial. Depress Anxiety.

[49] Bandelow B (2006). Defining response and remission in anxiety disorders: toward an integrated approach. CNS Spectr.

[50] Mataix-Cols D, Fernández de la Cruz L, Nordsletten AE (2016). Towards an international expert consensus for defining treatment response, remission, recovery and relapse in obsessive-compulsive disorder. World Psychiatry.

[51] Furukawa TA, Cipriani A, Barbui C (2005). Imputing response rates from means and standard deviations in meta-analyses. Int Clin Psychopharmacol.

[52] Posit team (2024). RStudio: integrated development environment for R.

[53] Sterne JAC, Savović J, Page MJ (2019). RoB 2: a revised tool for assessing risk of bias in randomised trials. BMJ.

[54] Peters JL, Sutton AJ, Jones DR (2010). Assessing Publication Bias in Meta-Analyses in the Presence of Between-Study Heterogeneity. J R Stat Soc Ser A Stat Soc.

[55] Sterne JAC, Becker BJ, Egger M, Rothstein HR, Sutton AJ, Borenstein M (2005). Publication bias in meta‐analysis.

[56] Rosenthal R (1979). The file drawer problem and tolerance for null results. Psychol Bull.

[57] Egger M, Smith GD, Schneider M (1997). Bias in meta-analysis detected by a simple, graphical test. BMJ.

[58] Duval S, Tweedie R (2000). Trim and fill: A simple funnel-plot-based method of testing and adjusting for publication bias in meta-analysis. Biometrics.

[59] Guyatt GH, Oxman AD, Vist GE (2008). GRADE: an emerging consensus on rating quality of evidence and strength of recommendations. BMJ.

[60] Hedges L, Olkin I (1985). Statistical Methods in Meta-Analysis.

[61] Harrer M, Cuijpers P, Furukawa TA (2021). Doing Meta-Analysis with R: A Hands-On Guide.

[62] Higgins JPT, Thompson SG, Deeks JJ (2003). Measuring inconsistency in meta-analyses. BMJ.

[63] Deeks JJ, Higgins JP, Altman DG (2019). Analysing data and undertaking meta-analyses. Cochrane Handb Syst Rev Interv.

[64] Borenstein M, Higgins JPT, Hedges LV (2017). Basics of meta-analysis: I^2^ is not an absolute measure of heterogeneity. Res Synth Methods.

[65] Schwarzer G, Carpenter JR, Rücker G (2015). Meta-Analysis with R.

[66] Hedges LV, Pigott TD (2001). The power of statistical tests in meta-analysis. Psychol Methods.

[67] Harrer M, Cuijpers P, Furukawa T (2019). Dmetar: companion R package for the guide “Doing meta-analysis in R.

