# Supplementary material for: Augmentation of cognitive-behavioural therapy for obsessive-compulsive and anxiety disorders: a protocol for a systematic review and meta-analysis
Source: BMJ Open. 2024 Nov 1;14(10):e090431. doi: 10.1136/bmjopen-2024-090431 (PMC11529745; doi:10.1136/bmjopen-2024-090431)
Supplement: online supplemental file 2 [file bmjopen-14-10-s002.pdf]

“Augmentation of cognitive-behavioral therapy for obsessive-compulsive and anxiety disorders: A protocol for a systematic review and meta-analysis”

Search syntaxes for all data bases

PubMed ____ 2

Embase via Ovid ____ 4

CINAHL via EBSCOhost ____ 6

PsycArticles, PsycInfo, PSYNDEX via EBSCOhost ____ 9

CENTRAL ____ 12

PubMed

("Obsessive-Compulsive Disorder"[Mesh]
OR "Panic Disorder"[Mesh]
OR "Phobic Disorders"[Mesh]
OR obsessive[tiab]
OR OCD[tiab]
OR anxiety[tiab]
OR anxious[tiab]
OR phobia[tiab]
OR phobic[tiab]
OR agoraphobia[tiab]
OR "panic disorder"[tiab])

AND

(augment*[tiab]
OR adjunct*[tiab]
OR adjuvant[tiab]
OR “add on”[tiab]
OR adding[tiab]
OR supplement*[tiab]
OR sequential[tiab]
OR enhanc*[tiab]
OR combin*[tiab]
OR boost*[tiab]
OR complement*[tiab]
OR additional[tiab]
OR conjunct*[tiab]
OR plus[tiab]
OR integrating[tiab]
OR integrate*[tiab])

AND

("Cognitive Behavioral Therapy"[Mesh]
OR "Cognitive Restructuring"[Mesh]
OR “cognitive behavio* therapy”[tiab]
OR CBT[tiab]
OR "cognitive behavio* group therapy"[tiab]
OR CBGT[tiab]
OR “cognitive therapy”[tiab]
OR “cognitive treatment”[tiab]
OR “cognitive restructuring”[tiab]
OR “behavio* treatment”[tiab]

OR “behavio* therapy”[tiab]
OR "exposure based"[tiab]
OR "exposure therapy"[tiab]
OR "EX RP"[tiab]
OR "exposure treatment"[tiab]
OR “exposure and response prevention”[tiab]
OR ERP[tiab])

AND

("Randomized Controlled Trial"[Publication Type]
OR "controlled trial"[tiab]
OR "clinical trial"[tiab]
OR RCT[tiab]
OR "parallel design"[tiab]
OR randomi*[tiab]
OR randomly[tiab]
OR "treatment trial"[tiab])

Note. We include textwords in title and abstract (tiab) and keywords with controlled vocabulary (MeSH terms).

Embase via Ovid

((exp anxiety disorder/ NOT exp post-traumatic stress disorder/)
OR obsessive.ti,ab.
OR OCD.ti,ab.
OR anxiety.ti,ab.
OR anxious.ti,ab.
OR phobia.ti,ab.
OR phobic.ti,ab.
OR agoraphobia.ti,ab.
OR "panic disorder".ti,ab.)

AND

(augment*.ti,ab.
OR adjunct*.ti,ab.
OR adjuvant.ti,ab.
OR "add on".ti,ab.
OR adding.ti,ab.
OR supplement*.ti,ab.
OR sequential.ti,ab.
OR enhanc*.ti,ab.
OR combin*.ti,ab.
OR boost*.ti,ab.
OR complement*.ti,ab.
OR additional.ti,ab.
OR conjunct*.ti,ab.
OR plus.ti,ab.
OR integrating.ti,ab.
OR integrate*.ti,ab.)

AND

((exp cognitive behavioral therapy/ NOT exp dialectical behavior therapy/)
OR exp exposure therapy/
OR "cognitive behavio* therapy".ti,ab.
OR "cognitive restructuring".ti,ab.
OR CBT.ti,ab.
OR "cognitive behavio* group therapy".ti,ab.
OR CBGT.ti,ab.
OR "cognitive therapy".ti,ab.
OR "cognitive treatment".ti,ab.
OR "behavio* treatment".ti,ab.
OR "behavio* therapy".ti,ab.
OR "exposure based".ti,ab.

OR "exposure therapy".ti,ab.
OR "EX RP".ti,ab.
OR "exposure treatment".ti,ab.
OR "exposure and response prevention".ti,ab.
OR ERP.ti,ab.)

AND

(exp randomized controlled trial/
OR "controlled trial".ti,ab.
OR "clinical trial".ti,ab.
OR RCT.ti,ab.
OR "parallel design".ti,ab.
OR randomi*.ti,ab.
OR randomly.ti,ab.
OR "treatment trial".ti,ab.)

Note. We include text words in title and abstract (.ti,ab.) and keywords with controlled vocabulary (Emtree terms).

CINAHL via EBSCOhost

(MH "Obsessive-Compulsive Disorder+"
OR MH "Generalized Anxiety Disorder"
OR MH "Social Anxiety Disorders"
OR MH "Panic Disorder"
OR MH "Phobic Disorders+"
OR TI obsessive
OR AB obsessive
OR TI OCD
OR AB OCD
OR TI anxiety
OR AB anxiety
OR TI anxious
OR AB anxious
OR TI phobia
OR AB phobia
OR TI phobic
OR AB phobic
OR TI agoraphobia
OR AB agoraphobia
OR TI "panic disorder"
OR AB "panic disorder")

AND

*(TI augment**
*OR AB augment**
*OR TI adjunct**
*OR AB adjunct**
OR TI adjuvant
OR AB adjuvant
OR TI "add on"
OR AB "add on"
OR TI adding
OR AB adding
*OR TI supplement**
*OR AB supplement**
OR TI sequential
OR AB sequential
*OR TI enhanc**
*OR AB enhanc**
*OR TI combin**
*OR AB combin**
*OR TI boost**

*OR AB boost**
*OR TI complement**
*OR AB complement**
OR TI additional
OR AB additional
*OR TI conjunct**
*OR AB conjunct**
OR TI plus
OR AB plus
OR TI integrating
OR AB integrating
*OR TI integrate**
OR AB integrate)*

AND

(MH "Cognitive Behavioral Therapy"
OR MH "Cognitive Restructuring"
OR TI "cognitive behavio therapy"*
OR AB "cognitive behavio therapy"*
OR TI CBT
OR AB CBT
OR TI "cognitive behavio group therapy"*
OR AB "cognitive behavio group therapy"*
OR TI CBGT
OR AB CBGT
OR TI "cognitive therapy"
OR AB "cognitive therapy"
OR TI "cognitive treatment"
OR AB "cognitive treatment"
OR TI "behavio treatment"*
OR AB "behavio treatment"*
OR TI "behavio therapy"*
OR AB "behavio therapy"*
OR TI "exposure based"
OR AB "exposure based"
OR TI "exposure therapy"
OR AB "exposure therapy"
OR TI "EX RP"
OR AB "EX RP"
OR TI "exposure treatment"
OR AB "exposure treatment"
OR TI "exposure and response prevention"
OR AB "exposure and response prevention"
OR TI ERP

OR AB ERP)

AND

(MH "Randomized Controlled Trials+"
OR TI "controlled trial"
OR AB "controlled trial"
OR TI "clinical trial"
OR AB "clinical trial"
OR TI RCT
OR AB RCT
OR TI "parallel design"
OR AB "parallel design"
*OR TI randomi**
*OR AB randomi**
OR TI randomly
OR AB randomly
OR TI "treatment trial"
OR AB "treatment trial")

Note. We include text words in title (TI) and abstract (AB) and keywords with controlled vocabulary (MH).

PsycArticles, PsycInfo, PSYNDEX via EBSCOhost

(DE "Obsessive Compulsive Disorder"
OR DE "Anxiety Disorders"
OR DE "Generalized Anxiety Disorder"
OR DE "Panic Attack"
OR DE "Panic Disorder"
OR DE "Phobias"
OR TI "panic disorder"
OR AB "panic disorder"
OR TI obsessive
OR AB obsessive
OR TI OCD
OR AB OCD
OR TI anxiety
OR AB anxiety
OR TI anxious
OR AB anxious
OR TI phobia
OR AB phobia
OR TI phobic
OR AB phobic
OR TI agoraphobia
OR AB agoraphobia
OR TI "panic disorder"
OR AB "panic disorder")

AND

*(TI augment**
*OR AB augment**
*OR TI adjunct**
*OR AB adjunct**
OR TI adjuvant
OR AB adjuvant
OR TI "add on"
OR AB "add on"
OR TI adding
OR AB adding
*OR TI supplement**
*OR AB supplement**
OR TI sequential
OR AB sequential
*OR TI enhanc**
*OR AB enhanc**

OR TI combin*
OR AB combin*
OR TI boost*
OR AB boost*
OR TI complement*
OR AB complement*
OR TI additional
OR AB additional
OR TI conjunct*
OR AB conjunct*
OR TI plus
OR AB plus
OR TI integrating
OR AB integrating
OR TI integrate*
OR AB integrate*)

AND

(DE "Exposure and Response Prevention Therapy"
OR DE "Exposure Therapy"
OR DE "Exposure and Response Prevention Therapy"
OR DE "Imaginal Exposure" OR DE "In Vivo Exposure"
OR DE "Virtual Reality Exposure Therapy"
OR DE "Cognitive Processing Therapy"
OR DE "Cognitive Techniques"
OR DE "Cognitive Therapy"
OR DE "Cognitive Hypothesis Testing"
OR DE "Cognitive Restructuring"
OR DE "Behavior Therapy"
OR DE "Behavior Therapy"
OR DE "Applied Behavior Analysis"
OR DE "Aversion Therapy"
OR DE "Behavior Modification"
OR DE "Cognitive Behavior Therapy"
OR TI "cognitive behavio* therapy"
OR AB "cognitive behavio* therapy"
OR TI CBT
OR AB CBT
OR TI "cognitive behavio* group therapy"
OR AB "cognitive behavio* group therapy"
OR TI CBGT
OR AB CBGT
OR TI "cognitive therapy"
OR AB "cognitive therapy"

OR TI "cognitive treatment"
OR AB "cognitive treatment"
OR TI "behavio treatment"*
OR AB "behavio treatment"*
OR TI "behavio therapy"*
OR AB "behavio therapy"*
OR TI "exposure based"
OR AB "exposure based"
OR TI "exposure therapy"
OR AB "exposure therapy"
OR TI "EX RP"
OR AB "EX RP"
OR TI "exposure treatment"
OR AB "exposure treatment"
OR TI "exposure and response prevention"
OR AB "exposure and response prevention"
OR TI ERP
OR AB ERP)

AND

(DE "Randomized Controlled Trials"
OR TI "controlled trial"
OR AB "controlled trial"
OR TI "clinical trial"
OR AB "clinical trial"
OR TI RCT
OR AB RCT
OR TI "parallel design"
OR AB "parallel design"
*OR TI randomi**
*OR AB randomi**
OR TI randomly
OR AB randomly
OR TI "treatment trial"
OR AB "treatment trial")

Note. We include text words in title (TI) and abstract (AB) and keywords with controlled vocabulary (DE).

CENTRAL

(obsessive
OR OCD
OR anxiety
OR anxious
OR phobia
OR phobic
OR agoraphobia
OR "panic disorder")

AND

(augment*
OR adjunct*
OR adjuvant
OR "add on"
OR adding
OR supplement*
OR sequential
OR enhanc*
OR combin*
OR boost*
OR complement*
OR additional
OR conjunct*
OR plus
OR integrating
OR integrate*)

AND

(cognitive NEXT behavio* NEXT therapy
OR "cognitive restructuring"
OR CBT
OR cognitive NEXT behavio* NEXT group NEXT therapy
OR CBGT
OR "cognitive therapy"
OR "cognitive treatment"
OR behavio* NEXT treatment
OR behavio* NEXT therapy
OR "exposure based"
OR "exposure therapy"
OR "EX RP"
OR "exposure treatment"

OR "exposure and response prevention"
OR ERP)

AND

("controlled trial"
OR "clinical trial"
OR RCT
OR "parallel design"
OR randomi*
OR randomly
OR "treatment trial")

Note. We include text words in title, abstract, and keywords.
