# Supplementary material for: Augmentation of cognitive-behavioural therapy for obsessive-compulsive and anxiety disorders: a protocol for a systematic review and meta-analysis
Source: BMJ Open. 2024 Nov 1;14(10):e090431. doi: 10.1136/bmjopen-2024-090431 (PMC11529745; doi:10.1136/bmjopen-2024-090431)
Supplement: Supplementary file 3 [file bmjopen-2024-090431.reviewer_comments.pdf]

PEER REVIEW HISTORY

BMJ Open publishes all reviews undertaken for accepted manuscripts. Reviewers are asked to complete a checklist review form and are provided with free text boxes to elaborate on their assessment. These free text comments are reproduced below.

ARTICLE DETAILS

Title (Provisional)

Augmentation of cognitive-behavioral therapy for obsessive-compulsive and anxiety disorders: A protocol for a systematic review and meta-analysis

Authors

Torbecke, Jonathan; Langhammer, Till; Lueken, Ulrike; Fendel, Johannes Caspar; Mewes, Lisa

---

VERSION 1 - REVIEW

**Reviewer**	**1**
**Name**	**Sharma, Eesha**
**Affiliation**	**National Institute of Mental Health and Neuro Sciences, Child And Adolescent Psychiatry**
**Date**	**11-Jul-2024**
**COI**	**None**

I have reviewed your protocol for a systematic review on augmentation of CBT for OCD and anxiety disorders. It is comprehensively put together, however, I have a few suggestions/ comments -

1. In the introduction please add a little more detail/ examples of the kind of augmentation strategies you intend to review. While at a couple of places attention, motivation and other strategies are listed, it would make sense to the reader if you can describe how are these strategies added on and how do they mechanistically augment CBT.

2. At a few places there are minor language errors in the introduction.

3. The running title "CBT-Augmentation for OCD and AD" is a little confusing as it could suggests CBT as the augmenting agent. Consider revising to - "Augmentation of CBT for OCD and AD".

**Reviewer**	**2**
**Name**	**Gosmann, Natan Pereira**

Affiliation **Universidade Federal do Rio Grande do Sul, Anxiety Disorders Outpatient Program**

Date **22-Jul-2024**

COI **None.**

This manuscript aims to report the protocol of a systematic review and meta-analysis designed to assess the efficacy of nonpharmacological augmentation strategies in addition to first-line cognitive behavioral therapy (CBT) for individuals diagnosed with anxiety disorders or obsessive-compulsive disorder (OCD). The manuscript is well-prepared and comprehensively written, and the publication of meta-analyses protocols is of great importance. However, there are some aspects that may be improved or better described, especially concerning the inclusion of relevant data and the statistical approach.

More specific comments are detailed below:

Rationale

1) Although the authors describe CBT as the first-line treatment for OCD and anxiety disorders, this is not a consensus among international guidelines, considering that selective serotonin reuptake inhibitors are also frequently recommended as first-line treatments. The term "first-line psychotherapeutic treatment," or a similar expression, would be a more appropriate description.

2) Considering that the authors describe estimating the effect of various augmentation strategies simultaneously as one of the strengths of this study, I suggest conducting a network meta-analysis. This approach would enable a proper comparison between interventions by considering both direct and indirect evidence.

3) The objective "1-What characterizes existing nonpharmacological augmentation strategies?" is unclear. I suggest rephrasing it to be more straightforward.

Methods

4) Throughout the manuscript, the authors use the term "population" when the more appropriate term would be "sample." I suggest a review of the usage of these technical terms, considering that they have conceptually different meanings.

5) The authors plan to select only one outcome measure per study; however, this decision would reduce statistical power and make the study vulnerable to potentially biased estimates due to specific characteristics of each included scale. For example, previous evidence indicates that clinician-rated scales tend to present higher estimates of placebo response in anxiety disorders compared to self-reported measures. I suggest including all available outcome measures in each study and considering a three-level approach, incorporating the study as an additional random variable to account for the statistical dependency between effect sizes from the same study.

6) Unpublished data will not be considered, which can lead to potentially biased results. I suggest considering the inclusion of unpublished sources even if the authors decide to perform a sensitivity analysis with only published studies.

7) Given that meta-analytic power depends on heterogeneity, total number of effect sizes, average number of participants per group, and the summary effect size, and that this research field may have some limitations concerning these aspects, I strongly suggest calculating statistical power for the meta-analysis a priori.

8) Risk of bias assessment: Cochrane does not have an explicit recommendation for how to perform an assessment of the overall for each study as planned by the authors. Therefore, I suggest considering a standardized method for the overall estimate of risk of bias, as reported by previous meta-analyses, and explicitly reporting which method will be used.

9) Given that some heterogeneity across studies is expected, odds ratios are preferred over risk ratios. Here is an excerpt from a study of interest for choosing effect size measures that explains why (Walter, S. D. (2000). Choice of effect measure for epidemiological data. Journal of Clinical Epidemiology, 53(9), 931-939.):

10) I suggest considering the analysis not only of efficacy and discontinuation rates, but also of rates of adverse events.

11) Sensitivity analysis will be performed regarding outliers. How are outliers going to be identified and how is this going to be applied for a sensitivity analysis?

12) The authors plan to conduct subgroup analyses and meta-regressions to explore the effect of at least nine potential moderators. According to Cochrane's recommendation of having at least ten outcome measures for each moderator, the authors would need at least 90 effect estimates, which is a rather optimistic expectation for this research field. Although the three-level approach would likely decrease the impact of this issue, it is important to report a prior plan for prioritizing specific moderators, preferably with reasonable rationale for this choice, to explore if the statistical power is insufficient to investigate all variables.

VERSION 1 - AUTHOR RESPONSE

Response to the decision letter

bmjopen-2024-090431 - "Augmentation of CBT for obsessive-compulsive disorder and anxiety disorders: A study protocol of a systematic review and meta-analysis"

Editor(s)' Comments to Author:

Please revise the ‘Strengths and limitations of this study’ section of your manuscript (after the abstract). This section should contain up to five short bullet points, no longer than one

sentence each, that relate specifically to the methods. The novelty, aims, results or expected impact of the study should not be summarised here.

Reply: We have revised the first point in the “Strengths and limitations of this study” section according to your recommendations. Here is the updated bullet point:

“This systematic review and meta-analysis of randomized controlled trials specifically assesses their impact on symptom reduction, response and dropout rates, focusing on nonpharmacological augmentation strategies of CBT for both obsessive-compulsive disorder and anxiety disorders.”

We hope that this revision meets your expectations and contributes to the improvement of our manuscript. Thank you again for your support and for highlighting these important aspects.

You have stated that you have used AI in preparation of your manuscript. As per our policy on AI use, please provide a transparent description of its use in your manuscript, including:

• What AI technology was used (the name of the technology)

• Why this AI technology was used (the reason for its use)

• How the AI technology was used (what the task of the technology was)

• Consider including a summary of the input, output, and the way in which the AI output was reviewed on the part of the authors as supplementary files or additional information for the editor to review. The editor may ask for more information and/or for information to be added to the content for internal use and/or for publication.

Reply: We appreciate your detailed feedback and the opportunity to clarify the use of artificial intelligence (AI) in the preparation of our manuscript.

We used ChatGPT for language editing to improve clarity, wording, and grammar. Here is a transparent description of its use:

1. AI Technology Used: We used ChatGPT, a language model developed by OpenAI.

2. Reason for use: The AI technology was employed to enhance the readability and linguistic quality of our manuscript, ensuring that the text is clear, well-structured, and grammatically correct.

3. How It Was Used: The AI technology was utilized to edit the manuscript’s language. Specifically, we provided sections of the text to ChatGPT, which then suggested improvements in wording, clarity, and grammar. These suggestions were carefully reviewed and, where appropriate, incorporated by the authors.

4. We used the following prompt to revise certain paragraphs in the manuscript: "Please revise the following paragraph for clarity, grammar, and adherence to scientific writing standards. Provide five alternative phrasings that maintain the original meaning while focusing on the following guidelines:

• Use active voice and prioritize verbs over nouns.

• Avoid unnecessary synonyms and keep the language concise.

• Minimize the use of negatives and forms of the verb 'to be'.

• Ensure the subject is close to the main verb to enhance readability."

We hope this clarifies the use of AI in our manuscript preparation and meets your requirements. Thank you again for your support and guidance.

Please include the planned start and end dates for the study in the methods section.

Reply: We included the following sentence in the methods section:

“The study is planned to commence in September 2024 and conclude in January 2025.”

Please insert the PROSPERO registration number.

Reply: Thank you for pointing out the need for the specific number to be included in the manuscript. We have now inserted the number as requested. The registration number is: CRD42024561027

Reviewer: 1

Dr. Eesha Sharma, National Institute of Mental Health and Neuro Sciences

Comments to the Author:

I have reviewed your protocol for a systematic review on augmentation of CBT for OCD and anxiety disorders. It is comprehensively put together, however, I have a few suggestions/ comments –

Reply: Thank you for your positive assessment and helpful comments. We have addressed each comment as thoughtfully as possible.

1. In the introduction please add a little more detail/ examples of the kind of augmentation strategies you intend to review. While at a couple of places attention, motivation and other strategies are listed, it would make sense to the reader if you can describe how are these strategies added on and how do they mechanistically augment CBT.

Reply: Thank you for this thoughtful comment. Your suggestion is particularly valuable as it pertains to the core aspect of our study.

Recent meta-analytical findings unfortunately provide little insights into how different augmentation strategies are added to CBT and their mechanisms of action. A recent meta-analysis on augmentation strategies for OCD [1] found that family involvement and motivational interviewing have shown promise, particularly in patients with more severe OCD and when administered separately. Another meta-analysis on attention bias modification as an augment in addition to CBT showed small effects on clinician-rated symptoms, particularly when administered in the same session as CBT [2].

We have taken your feedback seriously and clarified this aspect in the text. Specifically, we have added a more precise formulation of how augmentation strategies are added on and how the likely mechanistically augment CBT.

Nevertheless, the exact mechanisms by which these strategies augment CBT are still not fully understood. We hope to contribute to this understanding through our current study and provide more detailed information on how these augmentation strategies work.

The specific sections of the manuscript where these aspects have been clarified are listed below:

“Involving relatives of OCD patients in family therapy or groups likely improves family functioning (e.g., by reducing accommodation behaviors) enhancing the efficacy of CBT [3,4]. Motivational interviewing might enhance CBT outcomes by addressing patients ambivalence about treatment, boosting their self-efficacy, helping them confront anxiety during exposure

and response prevention exercises, clarifying the long-term relief from avoiding rituals, and supporting consistent completion of homework assignments [5,6]. While suggestions exist, the precise ways these strategies augment CBT are not yet fully understood."

"Hang et al. (2021) highlight that models by Beck and Clark [7] or Mogg and Bradley [8] both assume bottom-up (stimulus-driven) and top-down (cognitively driven) processes to contribute to anxiety. Therefore, combining CBT and ABM is promising because it involves both cognitive processes. Interestingly, this meta-analysis suggests that using ABM with CBT in the same session is more effective than applying them separately [2]."

Thank you once again for your constructive input, which has significantly improved the clarity of our manuscript.

2. At a few places there are minor language errors in the introduction.

Reply: Thank you very much for your valuable suggestion. We have carefully reviewed the introduction section and addressed minor language errors. We have made necessary corrections to improve clarity and readability. We hope that these revisions meet your expectations.

3. The running title "CBT-Augmentation for OCD and AD" is a little confusing as it could suggests CBT as the augmenting agent. Consider revising to - "Augmentation of CBT for OCD and AD".

Reply: We appreciate your valuable feedback and the clarification regarding the running title. We agree that the original title could be misleading and have revised it to "Augmentation of CBT for OCD and AD" as suggested.

Reviewer: 2

Dr. Natan Pereira Gosmann, Universidade Federal do Rio Grande do Sul

Comments to the Author:

This manuscript aims to report the protocol of a systematic review and meta-analysis designed to assess the efficacy of nonpharmacological augmentation strategies in addition to first-line cognitive behavioral therapy (CBT) for individuals diagnosed with anxiety disorders or obsessive-compulsive disorder (OCD). The manuscript is well-prepared and

comprehensively written, and the publication of meta-analyses protocols is of great importance. However, there are some aspects that may be improved or better described, especially concerning the inclusion of relevant data and the statistical approach.

More specific comments are detailed below:

Reply: Thank you very much for your helpful comments and suggestions. We greatly appreciate the time and effort you have taken to provide such detailed feedback. Your insights are extremely valuable and have been instrumental in improving the quality of our manuscript. We found your suggestions particularly relevant and helpful in enhancing the clarity and methodological rigor of our study.

Rationale

1) Although the authors describe CBT as the first-line treatment for OCD and anxiety disorders, this is not a consensus among international guidelines, considering that selective serotonin reuptake inhibitors are also frequently recommended as first-line treatments. The term "first-line psychotherapeutic treatment," or a similar expression, would be a more appropriate description.

Reply: We appreciate your valuable feedback and the thoughtful clarification regarding the description of CBT as a first-line treatment. We agree with your observation that selective serotonin reuptake inhibitors are also frequently recommended as first-line treatments according to various guidelines.

In response to your suggestion, we have revised the manuscript to more accurately reflect this context. The updated sentences are as follows:

“Besides serotonin reuptake inhibitors (SSRIs) cognitive behavioral therapy (CBT) is often referred to as the treatment of choice for OCD and ADs, with exposure methods and cognitive restructuring methods being applicable and commonly used to treat both disorders [9].”

“In line with these findings, national healthcare guidelines for the treatment of OCD and ADs recommend CBT as a first-line treatment besides SSRIs [10–12].”

2) Considering that the authors describe estimating the effect of various augmentation strategies simultaneously as one of the strengths of this study, I suggest conducting a network meta-analysis. This approach would enable a proper comparison between interventions by considering both direct and indirect evidence.

Reply: Thank you very much for your insightful comment and suggestion. We appreciate your recommendation to conduct a network meta-analysis, as it is indeed a statistically robust method that allows for the comparison of various interventions, even those that have not been directly compared in individual studies. This approach is methodologically sound and offers an interesting perspective.

However, the primary focus of our research is to determine whether there are generally effective and meaningful augmentation strategies and not to compare these strategies at this stage. Therefore, we have opted for a traditional meta-analysis in this initial step to establish a foundational understanding of their overall feasibility and efficacy.

Should the data reveal numerous effective augmentation strategies, we will certainly consider conducting a network meta-analysis as part of additional and exploratory post hoc analysis.

Thank you again for your valuable feedback.

3) The objective "1-What characterizes existing nonpharmacological augmentation strategies?" is unclear. I suggest rephrasing it to be more straightforward.

Reply: We agree with your suggestion and have revised the objective to be more precise. The updated objective is:

“1. What characterizes existing nonpharmacological augmentation strategies in terms of modality, content, mechanisms addressed, application methods, and intended health implications?”

We believe this formulation more accurately captures the aspects you highlighted.

Thank you once again for your valuable feedback and for helping us enhance the quality and rigor of our work.

Methods

4) Throughout the manuscript, the authors use the term "population" when the more appropriate term would be "sample." I suggest a review of the usage of these technical terms, considering that they have conceptually different meanings.

Reply: We sincerely appreciate your detailed feedback and the suggestion to review the usage of the terms “population” and “sample.” Your clarification is very helpful, and we agree that these terms have distinct conceptual meanings that should be used accurately.

In response to your comment, we have carefully reviewed all relevant sections of the manuscript and revised the terminology where necessary. The specific sections we addressed are listed below:

“Eligible study samples will include individuals who meet the diagnostic criteria of the Diagnostic and Statistical Manual of Mental Disorders (DSM) or the International Classification of Diseases (ICD) for OCD, panic disorder, agoraphobia, social anxiety disorder, generalized anxiety disorder, or specific phobia. We will include samples receiving no or consistent psychotropic medication. Therefore, samples with changes in their psychotropic medication during the trial will be excluded. We will also exclude trials involving non-clinical samples or undiagnosed patients.”

“We will extract information on (1) the study: authors, publication year, country, type of control, CBT-methods used, inclusion and exclusion criteria, (2) the sample: sample size at pre-, post- and follow-up measurement, age, sex, ethnicity, diagnosis, treatment resistance at baseline, comorbidity, medication, (3) the intervention and comparator: type of implemented augmentation strategy, delivery format, duration (timespan), duration of an average session, number of sessions, frequency of sessions, schedule (prior to, concurrently, or after CBT), use of measurement tools for intervention integrity including adherence to the protocol and to assess the clinical and program experience of the facilitating therapist, adverse events, and (4) the outcomes: measure of symptom severity, means and SDs of symptom severity of OCD and ADs at pre-, post- and follow-up measurement, response rates (including the definition used in the respective study and absolute numbers of response/non-response) and dropout rates (including the definition used in the respective study and absolute numbers of completing/discontinuing patients).”

“Sensitivity analyses will be performed regarding outliers, follow-up, study sample (intention-to-treat/completing participants), and risk of bias.”

5) The authors plan to select only one outcome measure per study; however, this decision would reduce statistical power and make the study vulnerable to potentially biased estimates due to specific characteristics of each included scale. For example, previous evidence indicates that clinician-rated scales tend to present higher estimates of placebo response in anxiety disorders compared to self-reported measures. I suggest including all available outcome measures in each study and considering a three-level approach, incorporating the study as an additional random variable to account for the statistical dependency between effect sizes from the same study.

Reply: Thank you very much for your valuable feedback and the thoughtful suggestion regarding the inclusion of multiple outcome measures. We appreciate your point that integrating different available outcome measures in a three-level approach would be statistically appropriate and strengthen the statistical power of our analysis.

We have considered this approach conceptually and deliberated on it intensively. However, we prefer to stick with clinician-rated outcome measures as they allow for better interpretability, especially given our focus on different anxiety disorders besides OCD. A cross-disorder measure for anxiety disorders like the Hamilton Anxiety Rating Scale (HAMA) is particularly useful in this context.

While the three-level approach offers a robust method for handling the dependencies between effect sizes within studies, it introduces significant complexity to the analysis. This added complexity can make it challenging to interpret the results, potentially obscuring rather than clarifying the relationships between variables. Moreover, if the primary goal of our research is to provide clear and actionable insights into the efficacy of augmentation of CBT for OCD and AD, the additional layers of complexity might detract from the practical impact of our findings. A simpler model may be more aligned with our objectives, ensuring that the conclusions are accessible and useful to both researchers and clinicians.

By focusing on clinician-rated outcome measures, we aim to avoid the complexities and potential inconsistencies that may arise from implementing various measures across different anxiety disorders. This focus helps maintain clarity and consistency in our analysis.

6) Unpublished data will not be considered, which can lead to potentially biased results. I suggest considering the inclusion of unpublished sources even if the authors decide to perform a sensitivity analysis with only published studies.

Reply: We deeply appreciate your thoughtful suggestion regarding the inclusion of unpublished sources to mitigate potential bias in our results. Your recommendation is indeed valuable and aligns with our goal of conducting a comprehensive and unbiased analysis.

To address this, we will contact the corresponding authors of all included studies to inquire whether they have or know of any additional unpublished data. Furthermore, we will reach out to authors of preregistrations (such as those on clinicaltrials.gov) and study protocols identified during our study search.

We have incorporated your suggested aspect into the manuscript as follows:

“To include potential unpublished data, we will contact the corresponding authors of all included studies to inquire whether they have or know of any additional unpublished data. Furthermore, we will reach out to authors of preregistrations (e.g. registered on clinicaltrials.gov) and study protocols identified during the search.”

Thank you once again for your insightful feedback and for helping us enhance the robustness of our study.

7) Given that meta-analytic power depends on heterogeneity, total number of effect sizes, average number of participants per group, and the summary effect size, and that this research field may have some limitations concerning these aspects, I strongly suggest calculating statistical power for the meta-analysis a priori.

Reply: Thank you for your insightful comment. Calculating the statistical power for a meta-analysis a priori is indeed important and can provide valuable insights into the robustness and reliability of the findings.

However, in our specific research context, we have decided not to conduct a priori power calculations. The primary reason is the lack of prior knowledge and detailed data in the

existing literature, which makes it challenging to estimate the necessary parameters accurately.

Since this research area is nascent, we lack crucial estimates for a power analysis, including expected effect size, effect size variance, and the number of studies to be included. Performing an a priori power analysis without reliable estimates would be speculative and ineffective.

Moreover, our focus is on conducting a comprehensive analysis of all available data to provide the most accurate summary of the evidence. This approach allows us to include all relevant studies, even if the data are incomplete or varied. Instead of relying on a priori power calculations, we plan to use adaptive strategies such as sensitivity analyses and subgroup analyses to explore the robustness of our findings and address any potential biases.

While we understand the importance of a priori power calculations, we believe that in our case, the benefits of a comprehensive and adaptive approach outweigh the potential advantages of pre-calculated statistical power.

Thank you again for your suggestion, and we hope this explanation clarifies our decision.

8) Risk of bias assessment: Cochrane does not have an explicit recommendation for how to perform an assessment of the overall for each study as planned by the authors. Therefore, I suggest considering a standardized method for the overall estimate of risk of bias, as reported by previous meta-analyses, and explicitly reporting which method will be used.

Reply: Thank you for your valuable comment. We recognize the importance of a standardized method for assessing overall risk of bias. We will use the Cochrane Risk of Bias (RoB 2) tool, which provides a standardized approach for evaluating the overall risk of bias for the outcome. Our study focuses on a unified measure that integrates both primary outcomes—symptom severity and response rates—while dropout rates are treated as a secondary outcome. Since dropout rates are simple counts rather than measured outcomes, domains related to outcome measurement and missing data hold less relevance. Furthermore, because dropout rates are reported as whole numbers, the risks of selective reporting or measurement bias are minimal. Thus, risk of bias assessments in this context may not offer meaningful insights specifically related to dropout rates. Given that our study's primary outcomes converge into a single comprehensive measure, we will continue to apply the overall risk of bias assessment for each outcome as outlined by the RoB 2 tool. This

method not only provides a detailed bias evaluation for each outcome but also, in our case, serves as a proxy for the overall study's risk of bias.

We hope this clarifies our approach and appreciate your understanding.

9) Given that some heterogeneity across studies is expected, odds ratios are preferred over risk ratios. Here is an excerpt from a study of interest for choosing effect size measures that explains why (Walter, S. D. (2000). Choice of effect measure for epidemiological data. Journal of Clinical Epidemiology, 53(9), 931-939.):

Reply: Thank you very much for your valuable feedback. We appreciate the important point you have raised regarding the preference for odds ratios over risk ratios, particularly in the context of heterogeneity across studies. We have revised the manuscript accordingly to reflect this consideration. The updated section is provided below.

"For the primary outcomes, we will calculate pooled Hedges' g [13] as a measure of the standardized mean difference in OCD/AD symptom severity at post-intervention as well as the pooled odds ratios for differences in response rates. For the secondary outcome, we will calculate pooled odds ratios for differences in dropout rates."

10) I suggest considering the analysis not only of efficacy and discontinuation rates, but also of rates of adverse events.

Reply: The consideration of adverse events is indeed an important aspect, and we appreciate the significance of the topic you have highlighted.

However, we acknowledge that adverse events are often a minor focus in many studies. Additionally, within the context of psychotherapy, the subject of adverse events is not yet clearly defined and outlined, which would make interpreting these results challenging.

To provide greater detail on this topic, we would like to reference the following studies:

Klatte, R., Strauss, B., Flückiger, C., & Rosendahl, J. (2023). Adverse events in psychotherapy randomized controlled trials: A systematic review. Psychotherapy Research, 1–16. https://doi.org/10.1080/10503307.2023.2286992

Vaughan, B., Goldstein, M. H., Alikakos, M., Cohen, L. J., & Serby, M. J. (2014). Frequency of reporting of adverse events in randomized controlled trials of psychotherapy vs. psychopharmacotherapy. Comprehensive Psychiatry, 55(4), 849–855. https://doi.org/10.1016/j.comppsych.2014.01.001.

Therefore, while we recognize the importance of your suggestion, we believe that including rates of adverse events in our analysis might not yield clear and interpretable results at this stage.

Thank you once again for your valuable feedback and for encouraging us to consider this important aspect.

11) Sensitivity analysis will be performed regarding outliers. How are outliers going to be identified and how is this going to be applied for a sensitivity analysis?

Reply: Your question addresses an important aspect of our analysis, and we appreciate the opportunity to clarify this point.

We will identify outliers using the non-overlapping confidence interval approach [14]. This approach will then be applied in our sensitivity analysis to assess the robustness of our results. Specifically, we will conduct the analysis both with and without the identified outliers to observe any significant changes in the overall findings.

We changed the relevant section in the manuscript, which is provided below:

"We will identify outliers using the "non-overlapping confidence interval approach" [14]."

12) The authors plan to conduct subgroup analyses and meta-regressions to explore the effect of at least nine potential moderators. According to Cochrane's recommendation of having at least ten outcome measures for each moderator, the authors would need at least 90 effect estimates, which is a rather optimistic expectation for this research field. Although the three-level approach would likely decrease the impact of this issue, it is important to report a prior plan for prioritizing specific moderators, preferably with reasonable rationale for this choice, to explore if the statistical power is insufficient to investigate all variables.

Reply: Thank you very much for your insightful comment and suggestion regarding the number of effect estimates needed for our planned subgroup analyses and meta-

regressions. According to Cochrane's recommendation, having at least ten effect estimates for each moderator is crucial for reliable results. We understand the concern about the potentially optimistic expectation of obtaining 90 effect estimates given the scope of our research.

To address this, we plan to utilize the same set of studies across multiple subgroup analyses where applicable. This approach is a common and accepted method in meta-analyses to maximize the use of available data while ensuring sufficient statistical power. We acknowledge that this strategy comes with certain dependencies and limitations, particularly in terms of interpretability. The potential overlap among studies in different subgroup analyses could introduce complexities in understanding the distinct effects of each moderator.

However, we believe that conducting these subgroup analyses is particularly important for gathering valuable information on which strategies are most effective within specific contexts.

Thank you again for your valuable feedback. We appreciate the significance of the topic you have highlighted.

References

1 Guzick AG, Cooke DL, Gage N, et al. CBT-Plus: A meta-analysis of cognitive behavioral therapy augmentation strategies for obsessive-compulsive disorder. J Obsessive-Compuls Relat Disord. 2018;19:6–14. doi: https://doi.org/10.1016/j.jocrd.2018.07.001

2 Hang Y, Xu L, Wang C, et al. Can attention bias modification augment the effect of CBT for anxiety disorders? A systematic review and meta-analysis. Psychiatry Res. 2021;299:113892. doi: 10.1016/j.psychres.2021.113892

3 Peris TS, Rozenman MS, Sugar CA, et al. Targeted Family Intervention for Complex Cases of Pediatric Obsessive-Compulsive Disorder: A Randomized Controlled Trial. J Am Acad Child Adolesc Psychiatry. 2017;56:1034-1042.e1. doi: https://doi.org/10.1016/j.jaac.2017.10.008

4 Thompson-Hollands J, Abramovitch A, Tompson MC, et al. A Randomized Clinical Trial of a Brief Family Intervention to Reduce Accommodation in Obsessive-Compulsive Disorder: A Preliminary Study. Behav Ther. 2015;46:218–29. doi: 10.1016/j.beth.2014.11.001

5 Merlo LJ, Storch EA, Lehmkuhl HD, et al. Cognitive-behavioral therapy plus motivational interviewing improves outcome for pediatric obsessive-compulsive disorder: A preliminary study. Cogn Behav Ther. 2010;39:24–7. doi: 10.1080/16506070902831773

6 Meyer E, Souza F, Heldt E, et al. A randomized clinical trial to examine enhancing cognitive-behavioral group therapy for obsessive-compulsive disorder with motivational interviewing and thought mapping. Behav Cogn Psychother. 2010;38:319–36. doi: https://doi.org/10.1017/s1352465810000111

7 Beck AT, Clark DA. An information processing model of anxiety: Automatic and strategic processes. Behav Res Ther. 1997;35:49–58. doi: 10.1016/S0005-7967(96)00069-1

8 Mogg K, Bradley BP. Anxiety and attention to threat: Cognitive mechanisms and treatment with attention bias modification. Behav Res Ther. 2016;87:76–108. doi: 10.1016/j.brat.2016.08.001

9 McKay D. Anxiety disorders. In: Norcross JC, VandenBos GR, Freedheim DK, et al., eds. APA handbook of clinical psychology: Psychopathology and health (Vol. 4). Washington: American Psychological Association 2016:61–95.

10 Deutsche Gesellschaft für Psychiatrie und Psychotherapie, Psychosomatik und Nervenheilkunde e.V. (DGPPN). S3-Leitlinie Zwangsstörungen – Langversion. AWMF. 2022. https://register.awmf.org/de/leitlinien/detail/038-017 (accessed 2 January 2024)

11 National Collaborating Centre for Mental Health (UK). Obsessive-Compulsive Disorder: Core Interventions in the Treatment of Obsessive-Compulsive Disorder and Body Dysmorphic Disorder. Leicester (UK): British Psychological Society (UK) 2006.

12 Deutsche Gesellschaft für Psychosomatische Medizin und Ärztliche Psychotherapie e.V. (DGPM). S3 - Leitlinie Behandlung von Angststörungen, Version 2. AWMF. 2021. https://register.awmf.org/de/leitlinien/detail/051-028 (accessed 2 January 2024)

13 Hedges L, Olkin I. Statistical Methods in Meta-Analysis. Academic Press 1985.

14 Harrer M, Cuijpers P, Furukawa TA, et al. Doing Meta-Analysis with R: A Hands-On Guide. 1st ed. Boca Raton: Chapman and Hall/CRC 2021.

VERSION 2 - REVIEW

Reviewer 2

Name Gosmann, Natan Pereira

Affiliation Universidade Federal do Rio Grande do Sul, Anxiety Disorders Outpatient Program

Date 20-Aug-2024

COI None.

This is the second review of this study, which aims to report the protocol of a systematic review and meta-analysis designed to assess the efficacy of nonpharmacological

augmentation strategies in addition to first-line cognitive behavioral therapy (CBT) for individuals diagnosed with anxiety disorders or obsessive-compulsive disorder (OCD). The authors have done an excellent job in their review, and almost all of my suggestions and concerns have been adequately addressed or implemented. Although not all of my suggestions were adopted, the authors clearly made a careful evaluation of their suitability for their study and provided satisfactory justifications when they decided not to implement them. I should only reiterate two of my comments related to statistical power, as I believe they could directly impact the reliability of the study when performed. Since this is a protocol and not necessarily design flaws of the project, these comments should be understood solely as suggestions for the authors and not necessarily as limitations that contraindicate the publication of the protocol.

Specific comments cited and numbered according to my first review:

Methods

7) Given that meta-analytic power depends on heterogeneity, total number of effect sizes, average number of participants per group, and the summary effect size, and that this research field may have some limitations concerning these aspects, I strongly suggest calculating statistical power for the meta-analysis *a priori*.

Reply: Thank you for your insightful comment. Calculating the statistical power for a meta-analysis a priori is indeed important and can provide valuable insights into the robustness and reliability of the findings.

However, in our specific research context, we have decided not to conduct a priori power calculations. The primary reason is the lack of prior knowledge and detailed data in the existing literature, which makes it challenging to estimate the necessary parameters accurately.

Since this research area is nascent, we lack crucial estimates for a power analysis, including expected effect size, effect size variance, and the number of studies to be included. Performing an a priori power analysis without reliable estimates would be speculative and ineffective.

Moreover, our focus is on conducting a comprehensive analysis of all available data to provide the most accurate summary of the evidence. This approach allows us to include all relevant studies, even if the data are incomplete or varied. Instead of relying on a priori power calculations, we plan to use adaptive strategies such as sensitivity analyses and subgroup analyses to explore the robustness of our findings and address any potential biases.

While we understand the importance of a priori power calculations, we believe that in our case, the benefits of a comprehensive and adaptive approach outweigh the potential advantages of pre-calculated statistical power.

Thank you again for your suggestion, and we hope this explanation clarifies our decision.

Reviewer comment: Although I understand that statistical power calculation is not yet widely implemented in meta-analyses, I should reiterate my suggestion to consider carrying out this calculation. Among the arguments presented by the authors for not including this analysis is the objective of conducting a comprehensive review, apparently assuming that a power calculation could somehow restrict data inclusion. Unlike sample size calculations for clinical trials, where researchers may limit the inclusion of individuals to the indicated sample size, in meta-analyses, the inclusion of studies should always be consistent with the inclusion criteria and search strategy (i.e., including all studies that meet the inclusion criteria), regardless of any statistical

power analysis. The difference would be that the meta-analytic estimates found would be more reliable if the parameters are similar to or more favorable than those used in the power calculation performed *a priori*. In this logic, I also respectfully disagree with the authors that performing this power calculation without precise parameters would be ineffective. Considering that the main goal of this type of method is to validate meta-analytic models that might face questions about their statistical power in the future (e.g., in a research field with a limited number of studies), it is quite acceptable to input into the model conservative/pessimistic parameters that are reasonable for this research field (i.e., few studies, small sample sizes, high heterogeneity). For the effect size expected, although there is no precise estimate of the expected effect size, quite reasonable alternatives would be to use the smallest effect size considered clinically relevant or effect sizes described for other augmentation strategies (Valentine JC, Pigott TD, Rothstein HR. How Many Studies Do You Need?: A Primer on Statistical Power for Meta-Analysis. Journal of Educational and Behavioral Statistics. 2010;35(2):215-247; Harrer, M., Cuijpers, P., Furukawa, T.A., & Ebert, D.D. (2021). Doing Meta-Analysis with R: A Hands-On Guide. Boca Raton, FL and London: Chapmann & Hall/CRC Press. ISBN 978-0-367-61007-4.).

12) The authors plan to conduct subgroup analyses and meta-regressions to explore the effect of at least nine potential moderators. According to Cochrane's recommendation of having at least ten outcome measures for each moderator, the authors would need at least 90 effect estimates, which is a rather optimistic expectation for this research field. Although the three-level approach would likely decrease the impact of this issue, it is important to report a prior plan for prioritizing specific moderators, preferably with reasonable rationale for this choice, to explore if the statistical power is insufficient to investigate all variables.

Reply: Thank you very much for your insightful comment and suggestion regarding the number of effect estimates needed for our planned subgroup analyses and meta-regressions. According to Cochrane's recommendation, having at least ten effect estimates for each moderator is crucial for reliable results. We understand the concern about the potentially optimistic expectation of obtaining 90 effect estimates given the scope of our research.

To address this, we plan to utilize the same set of studies across multiple subgroup analyses where applicable. This approach is a common and accepted method in meta-analyses to maximize the use of available data while ensuring sufficient statistical power. We acknowledge that this strategy comes with certain dependencies and limitations, particularly in terms of interpretability. The potential overlap among studies in different subgroup analyses could introduce complexities in understanding the distinct effects of each moderator.

However, we believe that conducting these subgroup analyses is particularly important for gathering valuable information on which strategies are most effective within specific contexts.

Thank you again for your valuable feedback. We appreciate the significance of the topic you have highlighted.

Reviewer comment: Even though the authors acknowledge Cochrane's recommendation of having at least ten effect estimates for each moderator, they report that they will address the potential issue of a limited number of studies for analyzing nine potential moderators by using the same set of studies across multiple subgroup analyses. In spite of that, it is unclear why this approach would not require the minimum of ten studies for each moderator assessed in the subgroup analyses (https://handbook-5-

1.cochrane.org/chapter_9/9_6_5_1_ensure_that_there_are_adequate_studies_to_justify.htm). Consistent with my previous comment, even the main analysis would need a power analysis to demonstrate adequate statistical power; therefore, it is certainly inaccurate to claim that the proposed subgroup analyses ensure adequate statistical power, as described by the authors in their response. Considering these limitations, it is also quite inaccurate to describe that these subgroup analyses would provide valuable information on which strategies would be more effective for different specific contexts. If the number of studies is insufficient for reliable subgroup analyses, I believe it would be more accurate to describe them as exploratory and hypothesis-generating for future research.

VERSION 2 - AUTHOR RESPONSE

Response to the decision letter

bmjopen-2024-090431.R1 - "Augmentation of CBT for obsessive-compulsive disorder and anxiety disorders: A study protocol of a systematic review and meta-analysis"

Reviewer: 2

Dr. Natan Pereira Gosmann, Universidade Federal do Rio Grande do Sul

Comments to the Author:

This is the second review of this study, which aims to report the protocol of a systematic review and meta-analysis designed to assess the efficacy of nonpharmacological augmentation strategies in addition to first-line cognitive behavioral therapy (CBT) for individuals diagnosed with anxiety disorders or obsessive-compulsive disorder (OCD). The authors have done an excellent job in their review, and almost all of my suggestions and concerns have been adequately addressed or implemented. Although not all of my suggestions were adopted, the authors clearly made a careful evaluation of their suitability for their study and provided satisfactory justifications when they decided not to implement them. I should only reiterate two of my comments related to statistical power, as I believe they could directly impact the reliability of the study when performed. Since this is a protocol and not necessarily design flaws of the project, these comments should be understood solely as suggestions for the authors and not necessarily as limitations that contraindicate the publication of the protocol. Specific comments cited and numbered according to my first review:

Reply: We greatly appreciate your thorough evaluation and constructive remarks, which have played a key role in strengthening the manuscript.

Methods

7) Given that meta-analytic power depends on heterogeneity, total number of effect sizes, average number of participants per group, and the summary effect size, and that this research field may have some limitations concerning these aspects, I strongly suggest calculating statistical power for the meta-analysis a priori.

Reply: Thank you for your insightful comment. Calculating the statistical power for a meta-analysis a priori is indeed important and can provide valuable insights into the robustness and reliability of the findings. However, in our specific research context, we have decided not to conduct a priori power calculations. The primary reason is the lack of prior knowledge and detailed data in the existing literature, which makes it challenging to estimate the necessary parameters accurately. Since this research area is nascent, we lack crucial estimates for a power analysis, including expected effect size, effect size variance, and the number of studies to be included. Performing an a priori power analysis without reliable estimates would be speculative and ineffective. Moreover, our focus is on conducting a comprehensive analysis of all available data to provide the most accurate summary of the evidence. This approach allows us to include all relevant studies, even if the data are incomplete or varied. Instead of relying on a priori power calculations, we plan to use adaptive strategies such as sensitivity analyses and subgroup analyses to explore the robustness of our findings and

address any potential biases. While we understand the importance of a priori power calculations, we believe that in our case, the benefits of a comprehensive and adaptive approach outweigh the potential advantages of pre-calculated statistical power. Thank you again for your suggestion, and we hope this explanation clarifies our decision.

Reviewer comment: Although I understand that statistical power calculation is not yet widely implemented in meta-analyses, I should reiterate my suggestion to consider carrying out this calculation. Among the arguments presented by the authors for not including this analysis is the objective of conducting a comprehensive review, apparently assuming that a power calculation could somehow restrict data inclusion. Unlike sample size calculations for clinical trials, where researchers may limit the inclusion of individuals to the indicated sample size, in meta-analyses, the inclusion of studies should always be consistent with the inclusion criteria and search strategy (i.e., including all studies that meet the inclusion criteria), regardless of any statistical power analysis. The difference would be that the meta-analytic estimates found would be more reliable if the parameters are similar to or more favorable than those used in the power calculation performed a priori. In this logic, I also respectfully disagree with the authors that performing this power calculation without precise parameters would be ineffective. Considering that the main goal of this type of method is to validate meta-analytic models that might face questions about their statistical power in the future (e.g., in a research field with a limited number of studies), it is quite acceptable to input into the model conservative/pessimistic parameters that are reasonable for this research field (i.e., few studies, small sample sizes, high heterogeneity). For the effect size expected, although there is no precise estimate of the expected effect size, quite reasonable alternatives would be to use the smallest effect size considered clinically relevant or effect sizes described for other augmentation strategies (Valentine JC, Pigott TD, Rothstein HR. How Many Studies Do You Need?: A Primer on Statistical Power for Meta-Analysis. Journal of Educational and Behavioral Statistics. 2010;35(2):215-247; Harrer, M., Cuijpers, P., Furukawa, T.A., & Ebert, D.D. (2021). Doing Meta-Analysis with R: A Hands-On Guide. Boca Raton, FL and London: Chapmann & Hall/CRC Press. ISBN 978-0-367-61007-4.).

Reply: We greatly appreciate your thoughtful and constructive feedback regarding the inclusion of a statistical power calculation in our meta-analysis. Following your recommendation, we have added a new section (2.7.2 A priori power calculation) to the manuscript, where we provide a detailed account of the a priori power analysis conducted using conservative parameters.

Please find the newly added section below:

“2.7.2 A priori power calculation

We conducted an a priori power analysis to evaluate the feasibility of detecting clinically meaningful effects within the expected constraints of our meta-analysis, including the small number of studies, limited sample sizes, and high heterogeneity. This allows us to better understand the potential limitations of our findings and ensures that our interpretations remain appropriately cautious. To address the difficulties of estimating precise values in this emerging field we decided to use conservative parameters. We aimed to detect a clinically relevant effect size of 0.2, setting the significance level at 0.05 for a one-sided test. Anticipating large heterogeneity across studies, we used a random-effects model and estimate the between-study variance following the guidelines by Hedges and Pigott [1] as suggested by Harrer et al. [2]. We expected to include at least 10 studies, each with at least 20 participants per group. The power analysis, conducted using the “power.analysis” function from the dmetar package [3] in RStudio [4], yielded an estimated power of 0.29. This suggests that, given these conservative parameters, the meta-analysis may have limited ability to detect small effects, reinforcing the need to interpret the findings cautiously.”

We believe this addition addresses your concerns by providing transparency around the statistical power of our meta-analytic estimates, especially given the complexities and limitations of the field. We appreciate your input, which has contributed significantly to the rigor of our analysis.

Thank you again for your valuable feedback.

12) The authors plan to conduct subgroup analyses and meta-regressions to explore the effect of at least nine potential moderators. According to Cochrane's recommendation of having at least ten outcome measures for each moderator, the authors would need at least 90 effect estimates, which is a rather optimistic expectation for this research field. Although the three-level

approach would likely decrease the impact of this issue, it is important to report a prior plan for prioritizing specific moderators, preferably with reasonable rationale for this choice, to explore if the statistical power is insufficient to investigate all variables.

Reply: Thank you very much for your insightful comment and suggestion regarding the number of effect estimates needed for our planned subgroup analyses and meta-regressions. According to Cochrane’s recommendation, having at least ten effect estimates for each moderator is crucial for reliable results. We understand the concern about the potentially optimistic expectation of obtaining 90 effect estimates given the scope of our

research. To address this, we plan to utilize the same set of studies across multiple subgroup analyses where applicable. This approach is a common and accepted method in meta-analyses to maximize the use of available data while ensuring sufficient statistical power. We acknowledge that this strategy comes with certain dependencies and limitations, particularly in terms of interpretability. The potential overlap among studies in different subgroup analyses could introduce complexities in understanding the distinct effects of each moderator.

However, we believe that conducting these subgroup analyses is particularly important for gathering valuable information on which strategies are most effective within specific contexts. Thank you again for your valuable feedback. We appreciate the significance of the topic you have highlighted.

Reviewer comment: Even though the authors acknowledge Cochrane's recommendation of having at least ten effect estimates for each moderator, they report that they will address the potential issue of a limited number of studies for analyzing nine potential moderators by using the same set of studies across multiple subgroup analyses. In spite of that, it is unclear why this approach would not require the minimum of ten studies for each moderator assessed in the subgroup analyses

(https://handbook-5-1.cochrane.org/chapter_9/9_6_5_1_ensure_that_there_are_adequate_studies_to_justify.htm). Consistent with my previous comment, even the main analysis would need a power analysis to demonstrate adequate statistical power; therefore, it is certainly inaccurate to claim that the proposed subgroup analyses ensure adequate statistical power, as described by the authors in their response. Considering these limitations, it is also quite inaccurate to describe that these subgroup analyses would provide valuable information on which strategies would be more effective for different specific contexts. If the number of studies is insufficient for reliable subgroup analyses, I believe it would be more accurate to describe them as exploratory and hypothesis-generating for future research.

Reply: Thank you for your detailed review and valuable comments on our proposed approach. We recognize that conducting subgroup and meta-regression analyses with a limited number of studies poses significant methodological challenges, particularly related to statistical power, independence, robustness, and validity.

To address these concerns, we have made changes to the relevant section in the manuscript, which now reads:

"If the number of studies available for the subgroup and meta-regression analyses is insufficient (i.e., fewer than ten times the number of subgroup and regression analyses), they will be conducted as exploratory and hypothesis-generating, rather than conclusive. This limitation will be carefully considered when interpreting the results, and the findings will be framed as preliminary insights to guide future research."

We will interpret the results cautiously to avoid overgeneralization. We hope these revisions address your concerns, and we greatly appreciate your constructive feedback.

References

1 Hedges LV, Pigott TD. The power of statistical tests in meta-analysis. Psychol Methods. 2001;6:203–17. doi: 10.1037/1082-989X.6.3.203

2 Harrer M, Cuijpers P, Furukawa TA, et al. Doing Meta-Analysis with R: A Hands-On Guide. 1st ed. Boca Raton: Chapman and Hall/CRC 2021.

3 Harrer M, Cuijpers P, Furukawa T, et al. dmetar: Companion R Package For The Guide "Doing Meta-Analysis in R." 2019.

4 Posit team. RStudio: Integrated Development Environment for R. 2024.

VERSION 3 - REVIEW

Reviewer 2

Name Gosmann, Natan Pereira

Affiliation Universidade Federal do Rio Grande do Sul, Anxiety Disorders Outpatient Program

Date 17-Sep-2024

COI None.

The authors have adequately addressed my comments and made the relevant revisions. I believe the current version of the protocol clearly expresses the robust methodology the authors intend to apply, and I wish them success in the execution of this project.

VERSION 3 - AUTHOR RESPONSE

Response to the decision letter

bmjopen-2024-090431.R2

Reviewer: 2

Dr. Natan Pereira Gosmann, Universidade Federal do Rio Grande do Sul

Comments to the Author:

The authors have adequately addressed my comments and made the relevant revisions. I believe the current version of the protocol clearly expresses the robust methodology the authors intend to apply, and I wish them success in the execution of this project.

Reviewer: 2 competing interests.: None.
